# Convolutional Neural Network-Based Robot Navigation Using Uncalibrated Spherical Images †

**DOI:** 10.3390/s17061341

**Published:** 2017-06-12

**Authors:** Lingyan Ran, Yanning Zhang, Qilin Zhang, Tao Yang

**Affiliations:** 1 School of Computer Science and Engineering, Northwestern Polytechnical University, Xi’an 710072, China; lingyanran@gmail.com (L.R.); tyang@nwpu.edu.cn (T.Y.); 2 Highly Automated Driving Team, HERE Technologies Automotive Division, Chicago, IL 60606, USA; samqzhang@gmail.com

**Keywords:** convolutional neural networks, vision-based robot navigation, spherical camera, navigation via learning

## Abstract

Vision-based mobile robot navigation is a vibrant area of research with numerous algorithms having been developed, the vast majority of which either belong to the scene-oriented simultaneous localization and mapping (SLAM) or fall into the category of robot-oriented lane-detection/trajectory tracking. These methods suffer from high computational cost and require stringent labelling and calibration efforts. To address these challenges, this paper proposes a lightweight robot navigation framework based purely on uncalibrated spherical images. To simplify the orientation estimation, path prediction and improve computational efficiency, the navigation problem is decomposed into a series of classification tasks. To mitigate the adverse effects of insufficient negative samples in the “navigation via classification” task, we introduce the spherical camera for scene capturing, which enables 360° fisheye panorama as training samples and generation of sufficient positive and negative heading directions. The classification is implemented as an end-to-end Convolutional Neural Network (CNN), trained on our proposed Spherical-Navi image dataset, whose category labels can be efficiently collected. This CNN is capable of predicting potential path directions with high confidence levels based on a single, uncalibrated spherical image. Experimental results demonstrate that the proposed framework outperforms competing ones in realistic applications.

## 1. Introduction

Vision-based methods have been attracting a huge amount of research interest for decades in autonomous navigation on various platforms, such as quadrotors, self-driving cars, and ground robotics. Various camera sensors and algorithms have been incorporated in these platforms to improve the machine’s sensing ability in challenging indoor and outdoor environments. For most applications, it is imperative to precisely localize the navigation path and detect potential obstacles. Among them, accurate position and orientation estimation is arguably the core task for mobile robot navigation.

**Figure 1:**
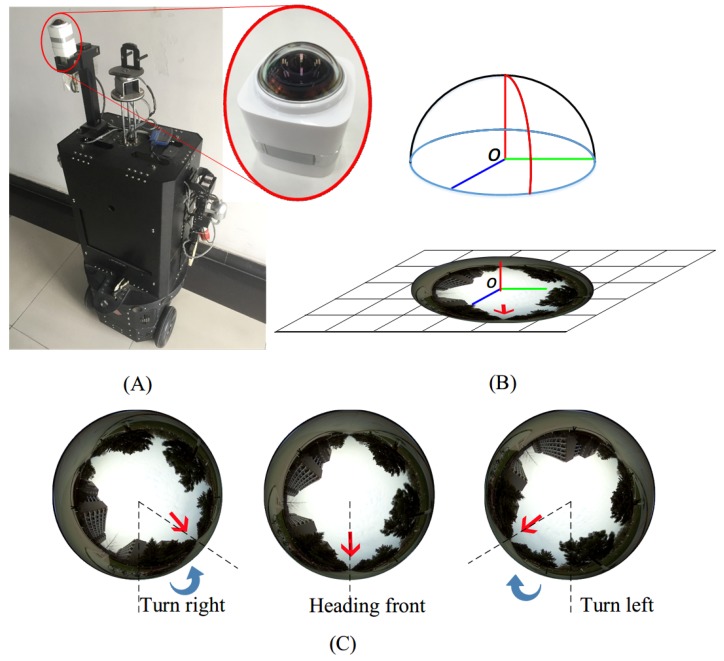
(**A**) A spherical camera mounted on a ground robot platform. (**B**) The spherical camera coordinate systems and its imaging model. Natural scenes are warped into the circular fisheye image. (**C**) Samples of captured spherical images. Red arrows denote the detected optimal path. Our objective is to generate navigation signals (denoted by blue arrows, i.e., steering direction and angles) based directly on these $360^{\circ }$ fisheye panoramas.

One major category of navigation methods, the simultaneous localization and mapping (SLAM), build virtual 3D maps of the surroundings while tracking the location and orientation of the platform. During last two decades, SLAM and its derivative methods have been dominating the navigation research field. Various systems have been proposed, such as MonoSLAM [1], PTAM [2], FAB-MAP [3], DTAM [4], KinectFusion [5], etc. Besides the use of monocular cameras, Caruso et al. [6] recently developed a SLAM system directly based on omnidirectional cameras and greatly expanded their applications. All the aforementioned SLAM systems share a common impediment to mobile platforms with limited computational capabilities, such as tablet PCs, quadrotors, and moving robotics as in our case (Figure 1).

Another category of human vision inspired approaches, i.e., the robot-oriented heading-field road detection and trajectory planning methods, address the navigation problem directly with visual paths detection via local road segmentation [7] and trajectory prediction [8], etc. For example, Lu et al. [9] built a robust road detection system based on hierarchical vision sensor. Chang et al. [10] presented a system using two biologically-inspired scene understanding models. In spite of their simplicity, the navigation performances of human vision inspired methods are heavily dependent on the quality of low level local features for their segmentation steps. In addition, for panorama images with heavy fisheye effect, traditional human vision inspired methods require prior calibration and warping preprocessing, further complicating these solutions.

In this paper, we focus on mobile robot navigation in open fields, with potential applications such as wheeled ground robots and low-flying quadrotors. This is a challenging problem: for one thing, such platforms lack computational capabilities required by SLAM-type algorithms and their typical tasks do not require sophisticated virtual 3D reconstruction. For another, those robots could be deployed outdoors with unpaved trails, where traditional path planning algorithms are likely to fail due to the increased difficulty in detecting unpaved surfaces.

As early as the 1990s, Pomerleau [11] formulated the road following task as a classification problem. Decades later, Hadsell et al. [12] developed a similar system for ground robot navigation in unknown environments. Recently, Giusti et al. [13] framed the camera orientation estimation as a three-class classification (Left, Front and Right) and captured a set of forest trail images with 3 head-mounted cameras, each pointing in one direction. Given one frame input, their model can decide the next optimal move (left/right turn or keep forward). One major drawback of this system is the coarse steering decisions, three fixed cameras and three choices are not precise enough for applications with higher orientation accuracy requirements.

We propose to replace multiple monocular cameras in [13] with a single spherical camera [14]. Thanks to the imaging characteristic of spherical cameras, each image captures the 360° panorama of the scene, eliminating the limitation on available steering choices.

One of the major challenges with spherical images is the heavy barrel distortion due to the ultra wide-angle fisheye lens, which complicates the implementation of conventional human vision inspired methods such as lane detection and trajectory tracking. Additional preprocessing steps such as prior calibration and dewarping are often required. However, in this paper, we circumvent these preprocessing steps by formulating navigation as a classification problem on finding the optimal potential path orientation directly based on the raw, uncalibrated spherical images.

Another challenge in the “navigation via classification” task is the shortage of negative training examples. Negative training samples typically represent wrong heading commands, which could lead to disasters such as collision. Inspired by [11], we reformulate navigation as classifying spherical images into a series of rotation categories. Unlike [11], negative training samples (wrong heading direction) could be conveniently generated by simple rotations of positive training samples (optimal heading direction), thanks to the $360^{\circ }$ fisheye panorama.

The contributions of this paper are as follows:

- A native “navigation via classification” framework based purely on $360^{\circ }$ fisheye panoramas is proposed in this paper, without the need of any additional calibration or unwarping preprocessing steps. Uncalibrated spherical images could be directly fed into the proposed framework for training and navigation, eliminating strenuous efforts such as pixel-level labeling of training images, or high resolution 3D point cloud generation for training.
- An end-to-end convolutional neural network (CNN) based framework is proposed, achieving extraordinary classification accuracy on our realistic dataset. The proposed CNN framework is significantly more computational efficient (in the testing phase) than SLAM-type algorithms and readily deployable on more mobile platforms, especially battery powered ones with limited computational capabilities.
- A novel $360^{\circ }$ fisheye panoramas dataset, i.e., the Spherical-Navi image dataset is collected, with a unique labeling strategy enabling automatic generation of an arbitrary number of negative samples (wrong heading direction).

The rest of this paper is organized as follows: Section 2 reviews related literature on deep learning based navigation and spherical images based navigation. Section 3 presents our proposed “navigation via classification” framework based directly on $360^{\circ }$ fisheye panoramas. A novel fisheye panoramas dataset (Spherical-Navi image dataset), is introduced in Section 4 together with the evaluation of the proposed “navigation via classification” framework in Section 5. Finally, Section 6 concludes this paper.

## 2. Background and Related Work

Numerous research efforts have been devoted to robot navigation since decades ago, and SLAM-type algorithms had been the preferable method until the recent trends in applying deep learning techniques in all low-level/mid-level computer vision tasks. Various classification methods (even with advanced and multisensory data, [15,16,17]) and radar based localization methods [18,19,20] had not been competitive enough against SLAM-type algorithms, due to increased sensor complexity and mediocre recognition accuracy. The “navigation via classification” framework is made both feasible and attractive to researchers only after deep learning based methods dramatically improved the classification accuracy.

The current advent of General-Purpose computing on Graphics Processing Units (GPGPU) reduces the typical CNN training time to feasible levels (the total training time of the proposed network is approximately 20 h). The low computational cost of deployed CNN makes real-time processing easily attainable (the proposed network prototype achieves 100 fps without any sophisticated optimization).

### 2.1. Deep Learning in Navigation

Deep learning has shown its overwhelmingly advantages over conventional methods in many research areas, including object detection [21,22] and tracking [23,24], image segmentation [25,26], and hyper-spectral image classification [27,28] etc. With the success of AlexNet [29] on ImageNet classification challenge [30], convolutional neural networks (CNNs) have become off-the-shelf solution for classification problems.

A number of improvements have been proposed over the years to further improve the classification performance of CNNs, such as the pioneering work [31], which shows the regularization efficiency of “Dropout”, especially for exploring extremely large amount of parameters. Another example is Lin et al. [32], which enhances model discriminability for local patches within the receptive field by incorporating micro neural networks within complex structures.

Navigation based on classifying the surrounding scene images with neural networks has been explored as early as 1990s. The Autonomous Land Vehicle In a Neural Network (ALVINN) [11] project is arguably one of the most influential ones, with realistic visual perception tasks and performance target of real-time processing. However, the tiny scale, oversimplified structure of early day neural networks, the primitive imaging sensors as well as abysmal computing power limited the usability of [11] in reality.

Subsequently, many improvements to ALVINN have been proposed. Hadsell et al. [12] developed a more stable system for navigation in unknown environments by incorporating a self-supervised learning framework capable of long-range sensing. This system is capable of accurately classifying complex terrains at distances up to the horizon (from 5 to over 100 m away from the platform, far beyond the maximum stereo range of 12 m), thus significantly improving path-planning.

Recently, Giusti et al. demonstrated a quadrotor platform autonomously following forest trails in [13]. They formulated the optimization of heading orientation as a three-class classification problem (Left, Front and Right) and captured a series of forest trail images with 3 inboard cameras, each facing Left, Front and Right, respectively. Given one image frame, the deployed CNN model determines the optimal heading orientation among the three available choices: left turn, straight forward or right turn. The major drawback of this design is the limited number of choices of three (due to three cameras), which is a compromise between steering accuracy and quadrotor load capacity.

### 2.2. Spherical Cameras in Navigation

There are a few published prior attempts on navigation based on spherical cameras, however, their performances are adversely affected by either rectification errors in pre-processing or lack of accurate reference frame.

First, considering the heavy barrel distortion due to the ultra wide-angle lens (e.g., omnidirectional cameras, fish-eye cameras, and spherical cameras), conventional navigation applications usually require pre-processing efforts such as calibration and rectification (i.e., removing fisheye effects). For example, Li [33] proposed a calibration method for full-view spherical camera images. We argue that this pre-processing steps incur unnecessary computational complexity and accumulate errors thus we favor the alternative approach, i.e., navigation based directly on spherical images.

A related but subtly different research field, spherical rotation estimation, has been investigated as early as a decade ago. For example, Makadia et al. [34,35] estimated 3D spherical rotations via the transformations induced in the spectral domain, and directly via the sphere images without correspondence, respectively. A recent paper by Bazin et al. [36] estimated spherical rotations based on vanishing points in omnidirectional images. Caruso et al. [6] proposed an image alignment method based on a unified omnidirectional model, achieving fast and accurate incremental stereo matching based directly on curvilinear, wide-angled images.

For the former “calibration and rectification” based methods, the error-accumulating pre-processing step would be eliminated if raw spherical images are directly used for navigation. For the latter group of methods, a major difference of these “spherical rotations estimation” attempts from the navigation tasks is the requirement of reference image frame: in rotation estimation problems, an estimated rotation angle is evaluated with respect to the reference image frame; however, reference image frames are almost never readily available in robot navigation applications. To overcome these limitations, a highly accurate, raw spherical image based “navigation via classification” framework is proposed in this paper.

## 3. CNN Based Robot Navigation Framework Using Spherical Images

Deep convolutional neural networks have been widely used in many computer vision and image sensing tasks, e.g., object detection [21,22], semantic segmentation [25], and classification [29,37,38]. In this section, a convolutional neural network based robot navigation framework is formulated to accurately estimate robot heading direction using raw spherical images. Figure 1 illustrates our capturing hardware platform, with a spherical camera mounted on a wheeled robot capable of capturing $360^{\circ }$ fisheye panoramas.

### 3.1. Formulation: Navigation via Classification

Given a series of *N* spherical images $x_{1},\cdots ,x_{N}$ sampled at time instances 1,⋯,N, respectively, the target of robot navigation can be formulated as the estimation of the optimal discrete heading direction ${\left\{y_{n}\right\}}_{n=1}^{N}\in Y$, with *Y* being some predefined turning options determined by robot tasks and complexity settings. Without loss of generality, let

(1) $$Y=\left\{Y_{0},Y_{\pm 1},\cdots ,Y_{\pm K}\right\}$$

where positive and negative *k* values represent anticlockwise and clockwise turn, respectively. Larger ∥k∥ values denote larger turning angles. The cardinality of *Y* (i.e., 2K+1, the number of navigation choices) could be conveniently set to satisfy the turning precision requirements of any given robot navigation application. Specifically, define $Y_{0}=0^{\circ }$ as the option to keep the current heading direction (straight forward).

With this model, the navigation task is the minimization of the global penalty *L* over the entire time instance range n=1,⋯,N,

(2) $$L=\sum_{n=1}^{N}\left\{1-\delta ({\hat{y}}_{n},y_{n})\right\}$$

in which ${\hat{y}}_{n}=F\left(x_{n};w,b\right)$ denotes network prediction at time instance *n* based on spherical image data $x_{n}$, where F(x;w,b) is a non-linear warping function learned with a convolutional neural network, *w* and *b* being the weights and bias terms, respectively. $y_{n}$ is the ground truth denoting the manually marked, optimal heading direction; and $\delta ({\hat{y}}_{n},y_{n})$ is the Kronecker delta,

(3) $$\delta \left({\hat{y}}_{n},y_{n}\right)=\left\{\begin{array}{cc} 0 & \mathrm{if}\;{\hat{y}}_{n}\neq y_{n},\\ 1 & \mathrm{if}\;{\hat{y}}_{n}=y_{n}. \end{array}\right.$$

### 3.2. Network Configuration and Training

**Figure 2:**
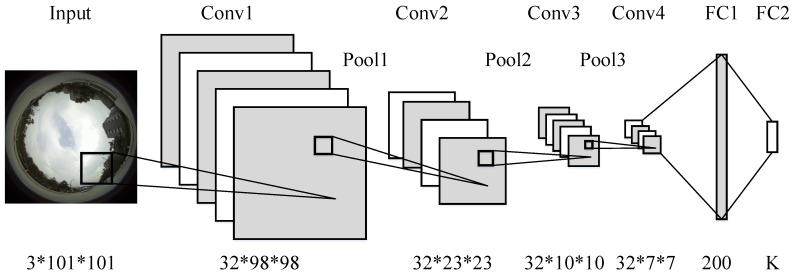
Proposed convolutional neural network based navigation framework. It consists of four convolutional layers, three pooling layers, and two fully connected layers. The inputs are raw, uncalibrated spherical images and the outputs are navigation signals (steering direction and angles).

Inspired by Alexnet [29] and Giusti et al. [13], a new convolutional neural network based robot navigation framework for the spherical images is proposed as shown in Figure 2.

Following the naming convention of current popular convolutional neural networks [13,29], convolutional layers (Conv), pooling layers (Pool) and fully connected layers (FC) are illustrated in Figure 2. The proposed network consists of four convolutional layers, three pooling layers, and two fully connected layers. Each convolutional layer is coupled with a max-pooling layer to enhance the local contrast.

Table 1 summarizes the network parameter settings of each layer of three networks, i.e., the baseline Giusti [13] network, the proposed “Rectified Linear Units” (ReLU) [39] based Model 1 network and another proposed “Parametric Rectified Linear Units” (PReLU) [40] based Model 2 network. Giusti et al. incorporated the scaled hyperbolic tangent activation function (Tanh) in [13] but did not provide the rationale behind this specific choice of non-linear warping function. In our experimental evaluation, we observe that both the ReLU based Model 1 network and the PReLU based Model 2 network outperform the Tanh units based one.

Optimizing a deep neural network is not trivial due to the gradient vanishing/exploding problem. In addition, it is also possible that optimization got stuck in a saddle point, resulting premature termination and inferior low level features. This becomes especially challenging for spherical images, due to their similar visual appearance.

To combat the aforementioned challenges, the Batch Normalization (BN) [41] is incorporated in the Model 2 network as shown in Table 1, which forces the network’s activations to generate larger variances across different training samples, accelerating the optimization in the training phase and also achieving a better classification performance.

During the training phase, both the Models 1 and 2 networks are optimized with the adaptive subgradient online learning (Adagrad) optimizer [42], allowing the derivation of strong regret guarantees. In addition, online regret bounds can be converted into a rate of convergence and generalization bounds. The usage of Adagrad optimization method eliminates the need of tuning the learning rates and momentum hyper-parameters as in the stochastic gradient decent (SGD) methods [43].

## 4. Spherical-Navi Dataset

A dataset with balanced class distributions is crucial for the effective training of a classification model. A common pitfall of navigation training dataset is the shortage of negative training samples. The negative samples typically represent wrong heading directions and could lead to accidents such as collision, hence they are not sufficiently collected in practice (to avoid damage to robot platform).

Inspired by [13], we propose to use spherical images for address this challenge. For one thing, every single spherical camera is capable of capturing a $360^{\circ }$ fisheye panorama, covering all possible heading directions, including wrong ones. For another thing, negative training samples could be conveniently generated by directly annotate the same $360^{\circ }$ fisheye panorama with an arbitrary number of wrong heading directions.

The following part of this section provides details on the proposed spherical image dataset, which is flexible with arbitrary number (2K+1 as in Equation (1)) of navigation choices (At each time instance *n*, heading directions $y_{n}=-Y_{K},\cdots ,0,\cdots ,Y_{K}$ are all potential navigation choices).

### 4.1. Data Formulation

**Figure 3:**
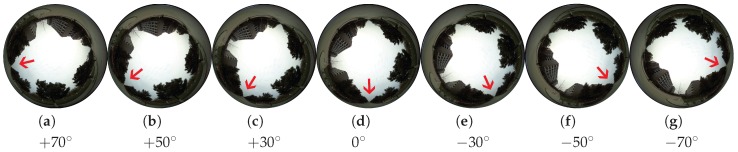
Sample images when an upward-facing spherical camera rotates. The central bottom pixels are the current heading direction of the robot platform and the red arrows represent one specific potential direction. The azimuth rotations of the robot platform merely lead to corresponding 2-dimensional rotations of the spherical images.

As shown in Figure 1B, an upward-facing spherical camera captures its $360^{\circ }$ surroundings and maps the scene into a non-rectilinear image. These spherical images share one distinctive characteristic, i.e., azimuth rotations of these cameras only lead to a simple two dimensional rotation of their captured images, as shown in Figure 3. The robot platform rotates from $+70^{\circ }$ to $-70^{\circ }$, the captured spherical images (Figure 3a–g) are corresponding 2-dimensional rotations of each other.

### 4.2. Data Capturing

**Figure 4:**
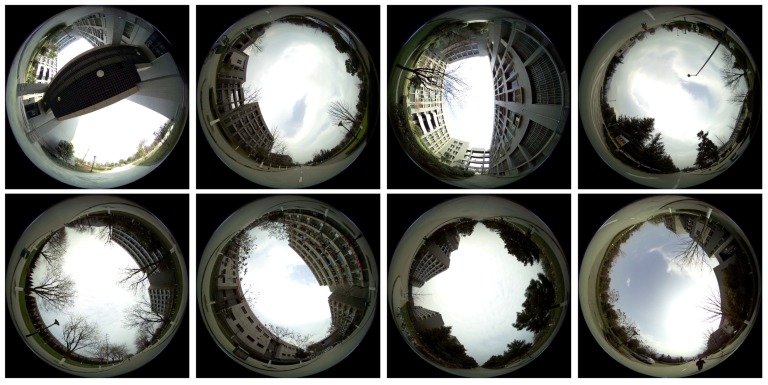
Sample images from the proposed Spherical-Navi dataset. Data collection is carried out throughout the school campus of Northwestern Polytechnical University, Xi’an, Shaanxi, China, and under different illumination conditions (i.e., different weather, time-of-day).

A robot platform shown in Figure 1A is used to collect images for training. The upward-facing spherical camera is mounted on top of the platform with a clearance of approximately 1.9 m above the ground, where the typical occlusions such as those caused by pedestrians and parked vehicles are rare. In total, we have captured ten video sequences with the robot platform traversing the school campus of Northwestern Polytechnical University, Xi’an, Shaanxi, China. The videos are captured at 60 frames per second with a resolution of 4608×3456. To increase the variety of the scenes in this dataset, navigation paths have been manually designed to cover as many feasible locations as possible. In addition, we also designed some overlapping path segments in these video sequences to discourage machine learning algorithms from simply “memorizing the routes”. Figure 4 shows typical example images in this dataset (Videos are publicly avaliable online at: https://hijeffery.github.io/PanoNavi).

### 4.3. Data Preparation

Due to the movement of the robot platform and lack of image stabilization, vibration could deteriorate a small fraction of video frames significantly. Therefore, the local temporal relative sharpness measurement $V_{i,p}$ [44] is incorporated to reject low quality image frames,

(4) $$V_{i,p}=\frac{\sum_{q\in N(p)}{∥\nabla J_{i,q}∥}_{1}}{\sum_{j=1}^{M}\sum_{q\in N(p)}{∥\nabla J_{j,q}∥}_{1}+\epsilon }$$

where $V_{i,p}$ is a normalized local sum of gradient magnitudes, with $J_{i}$ denoting *i*-th frame in a sequence of *M* frames, $J_{i,p}$ as its *p*-th pixel, N(p) as the set of spatially neighboring pixels of *p*, and the temporal relative sharpness of frame $J_{i}$ with *P* pixels is measured as the mean of local relative sharpness:

(5) $$V_{i}=\frac{\sum_{p=1}^{P}V_{i,p}}{P}$$

Additionally, temporal subsampling is carried out to reduce the very high temporal correlation between consecutive frames, since the video capturing frame rate is relatively high given the limited maximum speed of the robot platform. Without loss of generality, two video frames are randomly sampled (without replacement) per second (from the original 60 frames, less blurry frames that fail the Equation (5) criterion, if any). Six video sequences (with a total of 2000 frames) are randomly selected as training data; while the remaining 1500 frames from the other four video sequences are kept as testing data.

### 4.4. Label Synthesis

In the proposed “navigation via classification” framework, the optimal heading direction at time instance *n* is

(6) $$y_{n}\in \left\{-Y_{K},\cdots ,0^{\circ },\cdots ,Y_{K}\right\},$$

with $Y_{∥K∥}$ denoting the maximum steering angle (in degrees) permitted by the robot platform. In our experimental settings, $Y_{K}=90^{\circ }$ (anticlockwise turn) and $Y_{-K}=-90^{\circ }$ (clockwise turn). Collection of the positive labels (i.e., spherical images with correct heading direction) is trivial: manual inputs from the remote control are directly paired with the corresponding video frame. As is shown in Figure 3, azimuth rotations of an upward-facing spherical camera only lead to a planer rotation about the ground normal (*z* axis, which is perpendicular to the horizon). Therefore, negative label could be easily synthesized.

**Figure 5:**
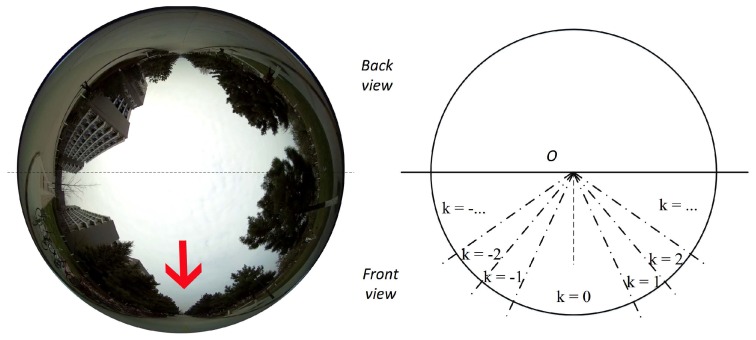
Negative Label Synthesis. Red arrow denotes the optimal heading direction, i.e., manual input from a remote control. Negative labels are rotations of this optimal heading direction, k=±1,⋯,±K. More image-label pairs are synthesized with small rotation angles (small ∥k∥ values) with respect to the optimal heading direction, in order to enhance the navigation “inertia”, i.e., to avoid frequent, unnecessary drastic steering adjustments.

After the robot platform has finished one capturing drive (without crashing) under manual remote control, the manual navigation inputs ${\left\{y_{n}\right\}}_{n=1}^{N}$ inputted by human are used directly as positive training labels (Positive training samples are image-label pairs denoting optimal heading direction, the label $y_{n}$ itself does not have a ’positive’ degree value. By definition in Equation (6), $y_{n}=Y_{0}=0^{\circ }$). More importantly, arbitrary number of negative samples could be conveniently synthesized at virtually no risk or cost at each time instance *n* by assigning alternative values to ${\left\{y_{n}\right\}}_{n=1}^{N}$. Figure 5 illustrates the synthesis of negative labels with various *k* values (k=±1,⋯,±K, larger ∥k∥ denotes larger offset from the optimal heading direction).

To minimize the dataset bias [45], most of the synthesized image-label pairs are sampled adjacent to the optimal heading direction (i.e., with small $Y_{∥k∥}$ values). In this way, the training set is statistically better matched with real navigation scenarios and empirically leads to a lower probability of consecutive contradictory steering decisions.

## 5. Experimental Results

### 5.1. Sky Pixels Elimination

The proposed robot platform collects data under various illumination conditions, due to different time-of-day and weather. Before feeding the spherical images into training networks, the central sky pixels (within a predefined radius) are masked out. Empirically, we found that these sky pixel values are heavily susceptible to illumination changes and our network gains 1%+ overall classification accuracy if these sky pixels are masked out. Subsequently, spherical images are normalized in the YUV color space to achieve zero mean and unit variance.

### 5.2. Network Setup and Training

Three algorithms are compared on the proposed Spherical-Navi dataset in Table 1. All of them share identical convolutional layers with the filter size 4. Their following pooling layers are of “Max-pooling” type, which select the local maximum values from the winning neurons.

**Figure 6:**
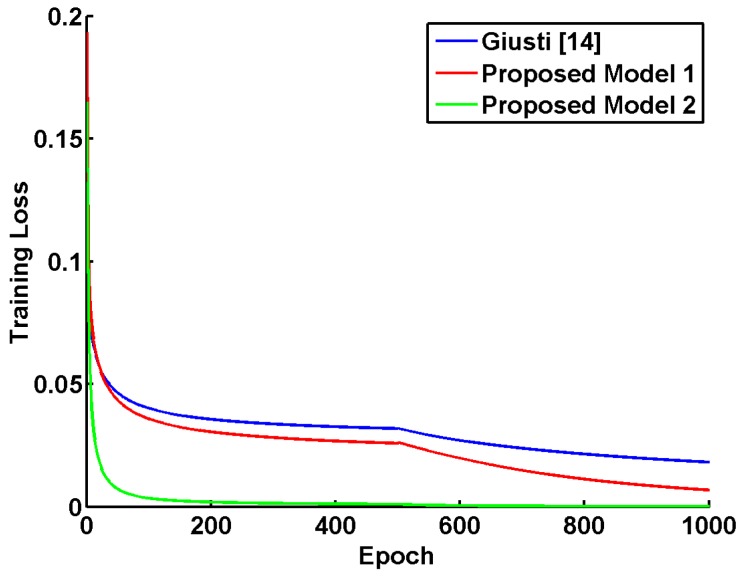
Training losses of competing models with the Adagrad optimizer. The learning rate is decreased by a factor of ten every 500 epochs. Our proposed “Model 2” with “Batch Normalization” achieves the fastest convergence and the lowest training loss.

We follow the training configurations in Giusti et al. [13], with weights initialized as in [40] and biases initialized by zeros. During the training procedure, a higher initial learning rate ($10^{-4}$) is selected for the proposed “Model 1” than that ($10^{-5}$) in the proposed “Model 2”. When the training loss stops decreasing, the learning rate is adjusted to one-tenth of the previous one. For better generalization to the testing phase, a mini-batch of size 10 is incorporated and all training samples are shuffled before each epoch. The training losses are illustrated against epoch in Figure 6, where our “Model 2” with batch normalization achieves significantly faster convergence to “better” local minima with smaller training loss value.

The proposed “Model 1” and “Model 2” algorithms are developed with the Torch7 deep learning package [46] and the respective network parameters are summarized in Table 1. With the proposed models and Spherical-Navi Dataset, all training procedures finish within 3 days using a PC with one Intel Core-i7 3.4 GHz CPU, or less than 20 h with a PC equipped with one Nvidia Titan X GPU. During the testing procedure, it takes the Nvidia Jetson TK1 installed onboard the robot platform only 10 milliseconds to process each spherical image.

### 5.3. Quantitative Results and Discussion

Table 2 summarizes the overall classification accuracies among competing algorithms with different number of navigation choices (i.e., 2K+1 as in Equation (1)). The LIBSVM [47] software with default settings (RBF kernel, C=1, γ=0.04) is chosen to implement the popular Support Vector Machine (SVM) classifier as a competing baseline. All deep learning based algorithms have achieved evident performance gains against the SVM baseline in various *K* settings. Generally, with more navigation choices (larger *K*), the classification accuracies drop for all competing algorithm, due to the increased complexity in the multiclass problem. Another factor that might contribute to imperfect classification is the camera mounting calibration, there could be some small rotating movements in the spherical camera during the capture process due to vibration.

**Figure 7:**
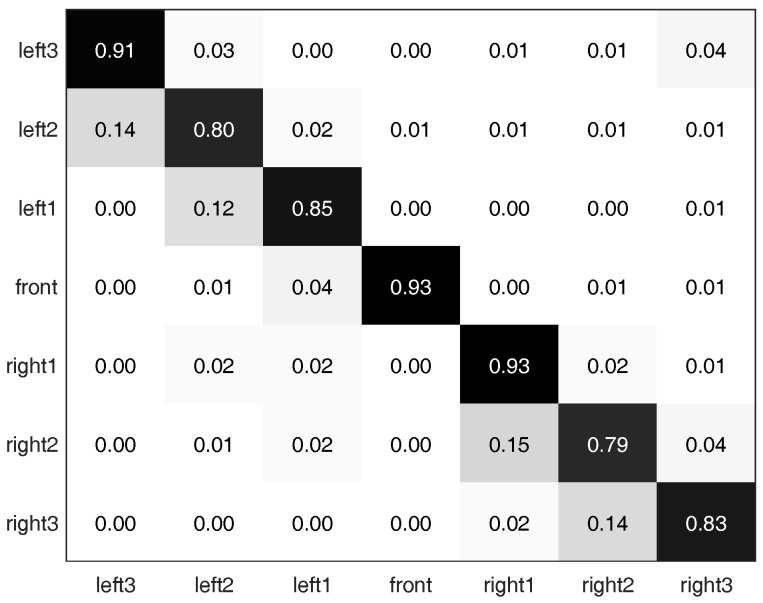
Classification confusion matrix filled with normalized accuracies in the case of 7 navigation choices. Diagonal elements denote correct classification; while off-diagonals denote mistakes.

Additionally, Figure 7 provides the multi-class classification confusion matrix [48,49] with 7 navigation choices (2K+1=7, last row in Table 2). With more navigation choices, spherical images from adjacent heading directions appear even more visually similar. The misclassification of adjacent choices leads to relatively larger sub-diagonal and super-diagonal values than other off-diagonal elements. We also note that while the robot platform is moving along a long stretch of straight path with non-distinctive scenes, Left3 view (leftmost view with $y_{n}$ perpendicular to drive path) appears to be a horizontal/vertical flip of Right3 view (rightmost view with $y_{n}$ perpendicular to drive path). This visual similarity could contribute to the slightly higher value in the upper-right element in the confusion matrix in Figure 7.

**Figure 8:**
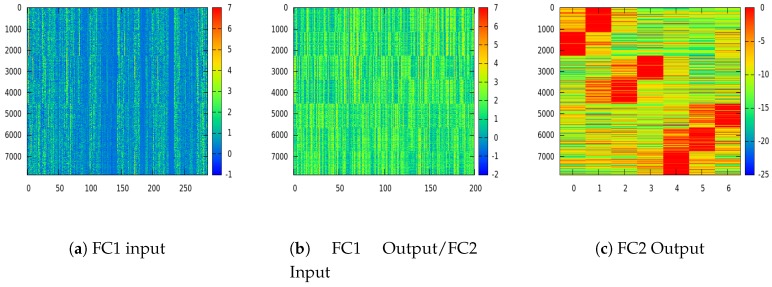
Extracted features from different layers in the proposed “Model 2” network with ordered 8000 sample training images and 7 navigation choices (X-axis: feature dimension; Y-axis: index of samples, best viewed in color). The network nonlinear mappings of fully connected layer 1 (FC1) ((**a**)→(**b**)) and FC2 ((**b**)→(**c**)) enhance the discriminability one after another.

Deep learning based methods can be generally regarded as a superbly discriminative feature extractor, and Figure 8 illustrates the progressive discriminability enhancement procedure layer after layer. Class-wise aggregated 8000 sample training images are fed into the proposed “Model 2” network with 7 navigation choices (K=3). The input of FC1 layer (i.e., the output from the last CONV layer), the output of FC1 layer (i.e., the input of FC2 layer) and the output of FC2 layer are visualized in Figure 8a–c, respectively. As the dimension of features decreases from 288 in “FC1 input” to 7 in “FC2 output” (Please refer to the last column in Table 1 for the layer structure of the proposed “Model 2” network), features are condensed into more compact and discriminative format, which is visually verifiable by inspecting the distinctive patterns in Figure 8c.

### 5.4. Robot Navigation Evaluation

The robot navigation performance of the proposed “Model 2” network (with 7 navigation choices) is evaluated in this section via multiple navigation tasks.

**Figure 9:**
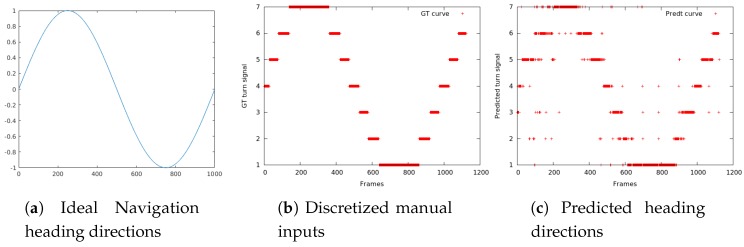
Robot Navigation Simulation. X-axis: frame index; Y-axis: sine of heading direction angle. The ideal, manually selected navigation path in (**a**) covers the entire possible heading direction angle $y_{n}$, from $0^{\circ }$ to $90^{\circ }$, then $-90^{\circ }$ and back to $0^{\circ }$. Therefore, $sin(y_{n})$ covers [−1,1]. (**b**) Limited by the 7 navigation choices, the robot navigation inputs are discretized. (**c**) visualizes the network predictions based purely on the collected spherical images.

In the first simulated evaluation, a special test drive path (covering all possible heading directions) is manually selected, visualized in Figure 9a and discretized in Figure 9b to match the number of available navigation choices. The robot platform (as shown in Figure 1A) is subsequently deployed with the exact navigation manual input in Figure 9b and a series of evaluation spherical images are collected correspondingly. These spherical images are then fed into the trained network as testing images and the predicted heading directions are visualized in Figure 9c.

The overall average prediction accuracy in Figure 9c is 87.3%, as compared to the ground truth in Figure 9b. Most of the misclassification errors happen during confusing the adjacent heading direction classes, which is understandable given the spatial similarity of typical scenes.

**Figure 10:**
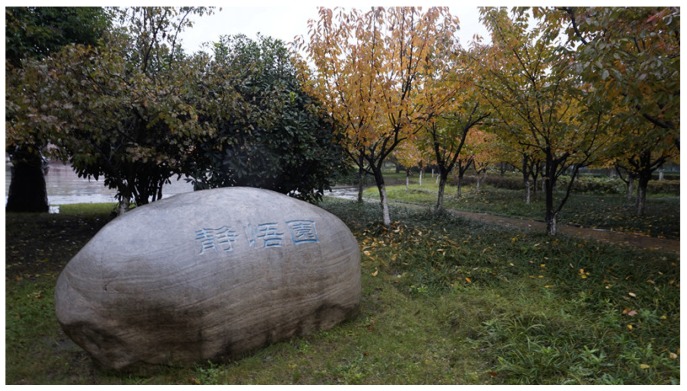
Jing-Wu Garden, inside the campus of Northwestern Polytechnical University, Xi’an, Shaanxi, China, where the navigation of the robot platform was tested.

In the following real-world navigation evaluation, the robot platform (as shown in Figure 1A) is deployed in the Jing-Wu Garden (as shown in Figure 10) inside the campus of Northwestern Polytechnical University, Xi’an, Shaanxi, China. The tested paths cover both paved walking trails and unpaved surfaces (mostly lawn).

**Figure 11:**
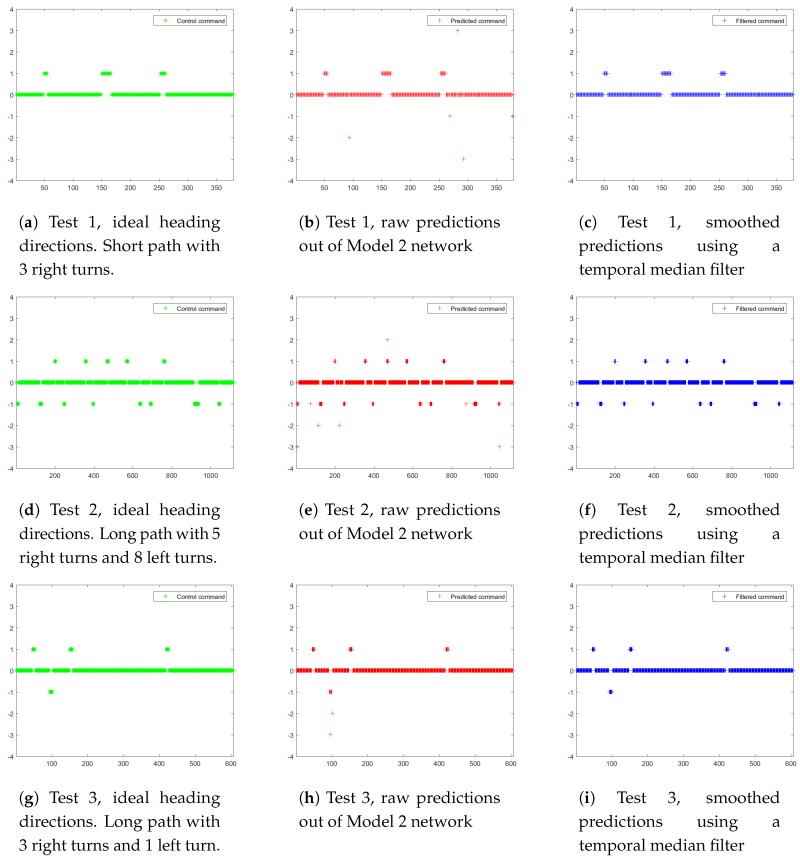
Robot navigation heading directions in 3 separate tests. X-axis: frame index; Y-axis: heading direction choices (0, ±1, ±2, ±3, positive values for right turns and negative values for left turns). (**a**,**d**,**b**) are manually labeled ideal heading directions; (**b**,**e**,**h**) are corresponding raw predictions from the proposed “Model 2” network; (**c**,**f**,**i**) are smoothed predictions using a temporal median filter of size 3.

**Figure 12:**
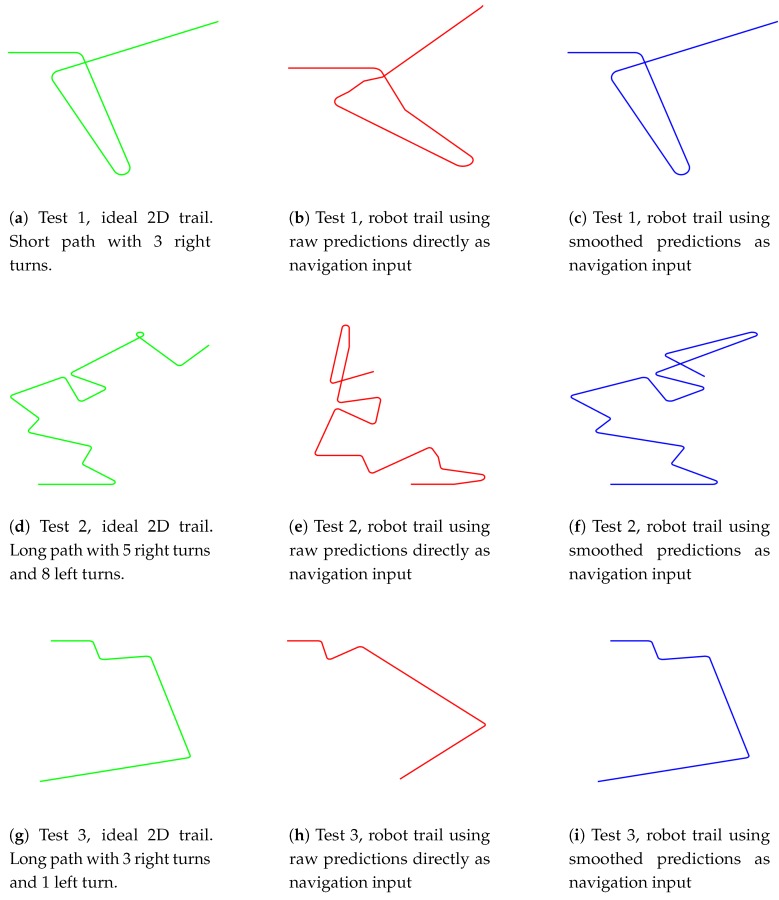
Robot navigation 2D trails in 3 separate tests. (**a**,**d**,**g**) are manually selected ideal drive trails; (**b**,**e**,**h**) are trails with raw network predictions as navigation inputs; (**c**,**f**,**i**) are trails with smoothed network predictions as navigation inputs.

3 separate tests (Test 1 is shown in Figure 11a–c and Figure 12a–c. Test 2 is shown in Figure 11d–f and Figure 12d–f. Test 3 is shown in Figure 11g–i and Figure 12g–i) are conducted under various road conditions in 3 phases:

1. Training data collection (Training data collection is illustrated in Figure 11a,d,g and Figure 12a,d,g): the robot platform is manually controlled to drive along 3 pre-defined paths multiple times, and the collected spherical images with synthesized optimal heading directions (detailed in Section 4.4) are used for “Model 2” network training.
2. Navigation with raw network predictions (Navigation with raw network predictions is illustrated in Figure 11b,e,h) and Figure 12b,e,h): the robot platform is deployed at the starting point of each trail and it autonomously navigate along the path with raw network predictions as inputs.
3. Navigation with smoothed network predictions (Navigation with smoothed network predictions is illustrated in Figure 11c,f,i) and Figure 12c,f,i): the robot platform is deployed at the starting point of each trail and it autonomously navigate along the path with smoothed network predictions as inputs. The smoothing is carried out with a temporal median filter of size 3.

The ideal heading directions and ideal two-dimensional trails are shown in Figure 11a,d,g and Figure 12a,d,g, respectively. A total of 7 heading direction choices are available at each frame, with 0 for straight forward, positive values for right turns and negative values for left turns. While navigating through corners, a series of consecutive small turning maneuvers (multiple +1 and −1 heading directions in Figure 11 and Figure 12) are preferred over sharp turns, allowing more training samples to be collected during these maneuvering frames.

Figure 11b,e,h demonstrate the raw predictions out of the “Model 2” network. Overall, vast majority of predictions are accurate for the straight forward (heading direction 0) sequences; while small portions of turning maneuvers are overestimated (with predicted heading directions ±2 and ±3). This could arise from the ambiguity of consecutive small turns and a single sharp turn achieving identical drive trail. In addition, there are only very subtle appearance differences during the limited number of frames while making turning maneuvers, which could result in confusions. In conjunction with the sporadic appearances of pedestrians, these confusions could lead to the spurious heading directions with excessive values (±2 and ±3). To remedy the situation, temporal coherence of heading directions need to be addressed. Empirically, a naive temporal median filter with window size 3 is effective enough to remove most spurious results, as shown in Figure 11c,f,i.

Figure 12 demonstrates the corresponding 2D trails of Figure 11. A few overestimated turning maneuvers in Figure 11b,e,h lead to wildly different trails (Figure 12b,e,h) from the ideal ones (Figure 12a,d,g). However, the smoothing-with-median-filtering remedy is highly successful in Figure 12c,i, only with Figure 12f showing an obvious difference from the Figure 12d towards the end of Test 2. A demonstration video is available online (Video demo: https://www.youtube.com/watch?v=4ZjnVOa8cKA.)

## 6. Conclusions

In this paper, a Convolutional Neural Network-based robot navigation framework is proposed to address the drawbacks in conventional algorithms, such as intense computational complexity in the testing phase and difficulty in collecting high quality labels in the training phase. The robot navigation task is formulated as a series of classification problems based on uncalibrated spherical images. The unique design of training data preparation eliminates time-consuming calibration and rectilinear correction processes, and enables automatic generation of an arbitrary number of negative training samples for better performance.

One potential improvement direction is the incorporation of temporal information via Recurrent Neural Networks (RNNs)/Long Short Term Memory networks (LSTMs). In addition, there are also multiple related problems for future research, such as indoor navigation and off-road collision avoidance. Source codes of the proposed methods and the Spherical-Navi dataset are available for download on our project web page (Project page: https://hijeffery.github.io/PanoNavi/). 

## Figures and Tables

**Table 1:** Comparsions of network structure and layer parameters of convolutional neural networks (CNNs).

	Features	Giusti [13]	Proposed Model 1	Proposed Model 2
0	3 × 101 × 101	Inputs	Inputs	Inputs
1	32 × 98 × 98	Conv	Conv	Conv
2	32 × 49 × 49	Pool	Pool	Pool
3	-	Tanh	ReLU	BN + PReLU
4	32 × 46 × 46	Conv	Conv	Conv
5	32 × 23 × 23	Pool	Pool	Pool
6	-	Tanh	ReLU	BN + PReLU
7	32 × 20 × 20	Conv	Conv	Conv
8	32 × 10 × 10	Pool	Pool	Pool
9	-	Tanh	ReLU	BN + PReLU
10	32 × 7 × 7	Conv	Conv	Conv
11	32 × 3 × 3	Pool	Pool	Pool
12	-	Tanh	ReLU	BN + PReLU
13	288→200	FC1	FC1	FC1
14	-	Tanh	ReLU	PReLU
15	200→K	FC2	FC2	FC2

**Table 2:** Classification Accuracies on the Spherical-Navi dataset.

Navigation Choices (i.e., 2K+1)	SVM [47]	Giusti [13]	Proposed Model 1	Proposed Model 2
3 choices (K=1)	84.03%	91.14%	92.64%	**94.30%**
5 choices (K=2)	78.92%	83.01%	84.82%	**93.07%**
7 choices (K=3)	70.30%	73.12%	72.36%	**87.73%**

## Abbreviations

The following abbreviations are used in this manuscript:

CNN	Convolutional Neural Networks
SLAM	Simultaneous Localization And Mapping
